# Genetic Drivers in Sebaceous Neoplasms: A Review of Germline and Somatic Mutations and Their Role in Treatment and Management Strategies

**DOI:** 10.3390/cancers17040659

**Published:** 2025-02-15

**Authors:** Christina Fujii, Ashley Mochizuki, Sandra Dreike, Joanne M. Jeter

**Affiliations:** City of Hope, Clinical Cancer Genomics, Center for Precision Medicine, Duarte, CA 91010, USA; cfujii@coh.org (C.F.); amochizuki@coh.org (A.M.); sdreike@coh.org (S.D.)

**Keywords:** sebaceous neoplasm, sebaceous carcinoma, Muir–Torre syndrome, Lynch syndrome, *MUTYH*-associated polyposis, microsatellite instability, mismatch repair

## Abstract

**Background**: The efficacy of germline testing in colorectal cancer has been proven; however, germline testing in individuals with sebaceous neoplasms is less well defined. This review aims to summarize the literature on sebaceous neoplasms to date, describing the somatic tumor profiles, tumor screening methods, and personal and family history that are suspicious of a germline mutation. Sebaceous neoplasms can be attributed to a variety of etiologies, including UV exposure, immunodeficiency, germline mutations, or multifactorial influences associated with aging. Sebaceous tumors with abnormal microsatellite instability and mismatch repair deficiency are indicative of a germline mutation in 20–50% of cases, which is similar to rates found in colorectal tumors. Personal and familial history can also be suggestive of a germline etiology in these patients and should be assessed routinely, as approximately 30% of individuals with sebaceous neoplasms carry a germline mutation. We outline a strategy for the identification of individuals at risk for germline mutations, recommendations for the management of mutation carriers, and treatment options for individuals with sebaceous neoplasms. **Conclusions**: Sebaceous tumors are most often sporadic; however, evaluations of a germline etiology are prudent to effectively identify those at risk of additional malignancies as well as at-risk family members. Referral to genetic counseling and germline genetic testing for individuals at risk can significantly impact cancer treatment and screening in patients and their families.

## Simple Summary

Approximately 30% of individuals with a sebaceous neoplasm are identified to have a germline mutation. The aim of our review is to identify germline and somatic mutations in sebaceous neoplasms and the steps for workup to determine those at the highest risk for a germline mutation. Early detection of a germline mutation is important for cancer treatment and screening and has medical implications for the family members of affected patients. Personal and family history are key components in risk assessments and should be included in the evaluation of all individuals diagnosed with sebaceous neoplasm. Genetic counseling is a valuable resource for those at risk for a germline mutation to determine appropriate testing strategies and to guide referrals to specialists for management if a mutation is identified.

## 1. Introduction

The term sebaceous neoplasms refers to a group of tumors with sebaceous differentiation, which includes sebaceous hyperplasia, sebaceomas, sebaceous adenomas, and sebaceous carcinomas [1]. These tumors can be divided into two subcategories: periocular and extraocular. These tumors, although most often sporadic, can be associated with inherited cancer conditions, notably Muir–Torre syndrome (MTS), a subtype of Lynch syndrome involving sebaceous carcinomas [1,2]. They have also been reported in individuals with *MUTYH*-associated polyposis (MAP), a genetic cancer predisposition syndrome that results in numerous colorectal polyps due to biallelic pathogenic variants in *MUTYH* [3,4]. In this review, we aim to describe sebaceous neoplasms and their associations with hereditary cancer syndromes, as well as the stratification and strength of these associations based on genes, tumor location, age of onset, and pathologic and familial features that may raise suspicion of these conditions.

## 2. Dermatologic Findings Associated with Muir–Torre Syndrome

### 2.1. Types of Sebaceous Neoplasms

#### 2.1.1. Sebaceous Hyperplasia

Sebaceous hyperplasia consists of benign skin lesions that occur when sebaceous glands in the skin collect an excess amount of sebum, forming small bumps on the skin at the base of hair follicles. About 1% of the population develops sebaceous hyperplasia in their lifetime, and it occurs more frequently in infants, the elderly, individuals who are immunosuppressed, and individuals with genetic predisposition to these neoplasms [5]. They commonly appear on the face and chest and typically match the color of the surrounding skin or are yellow or brown.

#### 2.1.2. Sebaceoma

Sebaceomas are benign cutaneous findings commonly found on the head and neck area of middle-aged to elderly individuals, with a median age of onset of about 70 years of age [6]. They mostly occur as solitary nodules or papules whose coloring may be yellow or match the color of the surrounding skin. Sebaceomas share many similarities with sebaceous adenomas, such as location, appearance, and age of onset, leading to challenges in differentiation. Sebaceomas are also commonly mistaken for basal cell carcinoma or trichoblastoma, given their partial sebaceous and at least 50% basaloid germ cell population.

#### 2.1.3. Sebaceous Adenoma

Sebaceous adenomas, compared to sebaceous hyperplasia, tend to be larger and often appear in clusters. Similarly to sebaceomas, they are benign neoplasms whose coloring may be yellow or match the color of the surrounding skin. They are typically located on the head and neck region, particularly the nose and cheeks. Middle-aged to elderly adults are more susceptible to these types of tumors. These benign tumors can be difficult to differentiate from other sebaceous neoplasms, particularly when the samples are small or the lesions are more superficial. Sebaceous adenomas are differentiated from sebaceomas in that the adenomas have a basaloid germinative cell population of less than 50% [1].

#### 2.1.4. Sebaceous Carcinoma

Sebaceous carcinoma is a rare skin cancer that arises from the sebaceous gland. The overall incidence in the United States between 2000 and 2016 was 2.4 cases per million [7]. Another study found the incidence varies by race, with 0.5 cases per million in the Black population, one case per million in the Asian and Pacific Islander population, and two cases per million in the White population [8]. In the United States, sebaceous carcinomas most commonly appear between the ages of 60 and 79, regardless of sex assigned at birth [7,8]. However, multiple studies have reported a higher incidence of sebaceous carcinoma, specifically of the eyelid, in individuals assigned female at birth compared to individuals assigned male at birth [7,9]. The diagnosis of sebaceous carcinomas can be challenging, but various pathologic features can aid in the exclusion of benign pathologies, such as sebaceomas and sebaceous adenomas [1]. Tumors that display a more infiltrative growth pattern, atypical mitoses, highly variable nuclei, and necrotic cell death are more likely to be sebaceous carcinomas [1]. Poorly differentiated sebaceous carcinomas can also be challenging to distinguish between basaloid squamous cell carcinomas, basal cell carcinomas, or Merkel cell carcinomas [1].

Sebaceous carcinomas are typically categorized as periocular or extraocular. Although 70–80% of sebaceous carcinomas occur on the head or neck, they can occur anywhere in the body where sebaceous glands are present [8,9]. The 5-year survival rate ranges between 70 and 97%, and the 10-year survival rate is approximately 79% [7,10]. One study found that periocular sebaceous carcinomas had a more favorable 5-year survival rate at 75% compared to extraocular occurrences at 68% [8]. Factors that can increase the risk of sebaceous carcinomas include immunosuppression, ultraviolet (UV) exposure, radiation exposure, and MTS [1].

- Periocular

Periocular tumors arise on the eyelid skin, which is the most common location for sebaceous carcinomas. Ocular tumors confined to the conjunctiva are extremely rare [11]. The upper eyelid is two to three times more frequently affected than the lower eyelid due to the high number of meibomian glands in this region. Women of Asian descent have been shown to be at greater risk for these neoplasms compared to other sebaceous neoplasms [12]. On the eyelid, they can present as a firm, round, enlarging nodule with inflammation and thickening of the eyelid. The diagnosis of periocular sebaceous carcinomas can be delayed due to their similar appearance to chalazion, conjunctivitis, blepharitis, or other inflammatory conditions of the eye [13,14]. Local spread is common and can involve the conjunctiva and cornea; advanced disease may even involve the orbit and adjacent structures [14]. Superficial biopsy should be performed for periocular tumors with dermatoscopic findings, including polymorphous atypical vessels, an underlying yellow background, and ulceration, which are consistent with sebaceous carcinomas [15].

b. Extraocular

Extraocular sebaceous carcinomas account for only 25% of all sebaceous carcinomas [16,17]. Extraocular tumors arise outside of the ocular region, most commonly in the head and neck, but they can occur in the trunk and extremities. They present as a red-yellow nodule or papule, often with ulceration. Extraocular tumors with suspicion of sebaceous carcinoma, as previously described for periocular tumors, should prompt thorough dermatoscopic evaluation and a deep biopsy, including the dermis [15].

### 2.2. Additional Skin Tumors Associated with MTS

#### Keratoacanthoma

Keratoacanthomas are benign, rapidly growing, dome-shaped skin tumors that begin in a hair follicle. They often occur on areas of the body that receive sun exposure, particularly the head, neck, arms, legs, and back of the hands. Clinically, they are almost indistinguishable when compared to squamous cell carcinomas and subsequently are often treated similarly. Keratoacanthomas are twice as likely to occur in males as opposed to females and are more common in individuals 60 years of age or older. Factors that can increase risk include immunocompromised status, hereditary cancer predisposition, such as MTS, UV radiation, exposure to carcinogenic chemicals, viral exposure, such as human papillomavirus (HPV), and trauma or surgery to the location [18].

## 3. Sporadic Etiologies and Molecular Profiles of Sebaceous Neoplasms

The molecular profile of sebaceous carcinomas has not been well defined, as limited studies have explored somatic genetic testing in these tumors. Tetzlaff et al. [19] performed whole exome next-generation sequencing on 27 sebaceous carcinomas (23 periocular and 4 extraocular) from 20 patients. The most common genes with somatic mutations identified in the periocular tumors were *TP53 (n* = 9), *RB1* (*n* = 6), *PIK3CA* (*n* = 2), *PTEN* (*n* = 2), *ERBB2 (n* = 2), and *NF1* (*n* = 2). Common mutations within the extraocular tumors were not easily defined due to the high tumor mutation burden attributed to microsatellite instability in three of the four tumors, but some of the genes with mutations identified in these tumors include the following: *ALK*, *BTK, FGFR2*, *PTEN*, *PDGFRB*, *MET*, *TSC1*, *ERBB2*, *PIK3R2*, *CHEK2*, *ABL2*, *HRAS*, *NF1*, and *ABL1*. Although the sample size of extraocular tumors was small in this study, Kruskal–Wallis testing identified a significant difference between the number of mutations in extraocular compared to ocular sebaceous carcinomas (*p* = 0.036), with more mutations detected in the extraocular lesions. A separate study in Korea examined 29 sebaceous carcinomas (20 periocular, 9 extraocular) and found similar results to Tetzlaff et al., where the most common genes with mutations in periocular tumors were *TP53* (*n* = 13) and *RB1* (*n* = 3) [20]. The most common mutation in extraocular tumors was *NOTCH1* (*n* = 5), which was significantly enriched compared to periocular tumors (*p* = 0.001).

Additional studies have investigated the somatic profiles of tumors in the setting of risk factors suspected to increase the risk of sebaceous carcinomas. Three categories of somatic profiles emerged: tumors associated with UV radiation, immunosuppression, and multifactorial influences. In the United States, 73% of sebaceous carcinoma cases occur on the head and neck, areas that are often exposed to the sun [21]. In addition, 85% of these cases occur in White individuals, who have the increased risk factor of less photoprotective melanin pigment in their skin compared to other racial groups. When ambient UV radiation exposure was assessed via geographic location, there was an increased risk for sebaceous carcinoma both with and without MTS [21]. These data support UV radiation as a potential etiology for sebaceous carcinomas. The somatic profile of these UV-related tumors differs from that of their mismatch repair counterparts. Sebaceous neoplasms associated with UV radiation have a higher tumor mutational burden, are more likely to be poorly differentiated and infiltrative, and have frequent *TP53*, *NOTCH1/2*, *FAT3*, *RREB1*, and *KMT2D* mutations, a profile resembling that of cutaneous squamous cell and basal cell carcinomas [22].

Immunosuppression has also been noted as a risk factor for sebaceous carcinomas. Lanoy et al. found that the risk of sebaceous carcinomas was 8-fold higher in individuals with acquired immunodeficiency syndrome [23]. Similarly, individuals who are receiving immunosuppressive therapy for solid organ transplants have an estimated 25- to 34-fold increased risk compared to the general population [24,25]. Tumors in individuals with immunosuppression are less likely to have common driver mutations, as shown in a study of a group of 29 patients, in which 31% (*n* = 9/29) lacked mutations in *TP53*, *RB1*, or *NOTCH1/2* [26]. In four of these nine tumors (44%), the patients were HPV-positive, and the age of onset tended to be younger and without local recurrence [26].

Lastly, there is a population of sebaceous neoplasms that have a low mutation burden, unlike those associated with UV radiation, that are likely attributed to multifactorial influences, such as aging. Cutaneous sebaceous carcinomas with a low mutational burden often occur on the head and neck, as is typical, but lack recurrent characteristic mutation profiles on somatic testing [22]. Ocular sebaceous carcinomas with this low mutational burden often have *ZNF750* mutations, with some patients also having *TP53* or *RB1* driver somatic mutations, and other patients having an increased risk of developing these tumors due to immune deficiency [22]. Overall, in addition to mismatch repair deficiency driving the formation and development of sebaceous neoplasms, which is described in greater detail within successive sections, there are alternative risk factors, including exposure to UV radiation, immunosuppression, and multifactorial influences in sporadic cases that should be considered when assessing the etiology of a sebaceous neoplasm. These alternative factors may aid in determining an etiology for a patient’s personal and/or family history of sebaceous neoplasias in the absence of an identifiable germline variant.

## 4. Mismatch Repair Deficiency and Microsatellite Instability in Sebaceous Neoplasms

### 4.1. MMR Deficiency

Mismatch repair (MMR) proteins associated with Lynch syndrome include *MLH1*, *MSH2*, *MSH6*, and *PMS2*—all of which are essential components in DNA repair. The MMR proteins interact as heterodimers that detect and repair mismatches in DNA, such as inaccurately paired bases and DNA abnormalities caused by insertions or deletions. The pairings of heterodimers are as follows: *MLH1* binds with *PMS2*, and *MSH2* binds with *MSH6*. *MLH1* and *MSH2* are key proteins within the heterodimer that can bind with other proteins to perform DNA mismatch repair. However, *PMS2* and *MSH6* proteins only dimerize with *MLH1* and *MSH2*, respectively. Therefore, the absence of *MLH1* or *MSH2* expression will also cause absent protein expression of the corresponding dimerized protein. This is an essential concept in interpreting MMR protein expression and the likelihood of a Lynch syndrome mutation. The expression of MMR proteins in a tumor is detected using immunohistochemistry (IHC) staining [27]. Abnormal MMR protein function within a tumor, referred to as mismatch repair deficiency (dMMR), may lead to tumor microsatellite instability (MSI) due to the cell’s inability to perform DNA mismatch repair.

### 4.2. MSI

Microsatellites are repetitive DNA sequences, typically made up of one to six base pairs, which may repeat up to 100 times throughout the genetic code. Highly repetitive sequences are prone to strand slippage during DNA replication, resulting in errors. Tumors with dMMR typically result in MSI due to the cell’s inability to repair errors. MSI in a tumor is detected using a methodology combining polymerase chain reaction with fluorescent primers and capillary electrophoresis [28]. The standard approach to MSI testing utilizes a panel of microsatellite markers on the National Cancer Institute (NCI) microsatellite panel (two mononucleotide—BAT25/26; three dinucleotide markers—D2S123, D5S346, and D17S250) and compares these markers with a paired normal sample to identify high microsatellite instability (MSI-H) within the tumor [29]. The presence of instability in at least two markers is considered MSI-H, instability in one marker is considered low microsatellite instability (MSI-L), and stability in all markers would indicate microsatellite stability (MSS) [29]. Additional testing may be necessary to exclude MSI-L tumors, especially in the setting of only aberrant dinucleotide repeat mutations.

### 4.3. Tumor Screening with MMR-IHC and MSI

Universal tumor screening with MMR-IHC and MSI is recommended for all colorectal tumors at the time of diagnosis [30]. This is due to the relatively high rates of germline Lynch syndrome mutations in individuals with dMMR staining (25–67%); however, a common alternative etiology for dMMR involving *MLH1* and *PMS2* is sporadic *MLH1* promoter methylation [31,32]. There is notably a lower sensitivity and specificity for Lynch syndrome in individuals with dMMR by MMR-IHC in extraocular sebaceous carcinomas (81–85% sensitivity and 48% specificity) versus in colonic tumors (92–94% sensitivity and 88–100% specificity) [33]. Limited studies have explored MMR-IHC and MSI testing sensitivity in sebaceous carcinomas, and there are debates regarding the use of these screenings in sebaceous carcinomas, as their clinical relevance is not well defined.

Historically, studies have explored tumor screening with MSI and MMR-IHC in individuals with sebaceous and internal tumors concerning MTS. Machin et al. [34] examined tumor samples from six patients with internal Lynch-associated tumors and cutaneous tumors and found that all ten cutaneous (eight sebaceous adenomas, one sebaceous carcinoma, and one keratoacanthoma) and all eleven internal Lynch-associated tumors exhibited MSI. dMMR on MMR-IHC was concordant for all tumors in this study, with the majority exhibiting loss of *MSH2* protein expression (*n* = 5). This result is suggestive of a germline etiology for these tumors. Entius et al. [35] compared MSI in tumors from 13 patients with MTS and 8 patients with sporadic sebaceous carcinoma. MSI was detected in 69% (*n* = 9/13) of the patients with MTS, while none of the sporadic sebaceous carcinoma tumors exhibited MSI (*p* = 0.002). MMR-IHC for *MLH1* and *MSH2* was concordant in 89% (*n* = 8/9) of tumors with MSI: four had loss of *MLH1* and four had loss of *MSH2*. These studies demonstrate the potential use of testing individuals for germline pathogenic variants in the setting of a sebaceous carcinoma with MSI.

More recent studies focused on identifying dMMR in an unselected population of sebaceous neoplasms. Notably, very few of these studies explored MSI status in these tumors (Table 1). Cook et al. [2] identified that 32% of sebaceous carcinomas in their cohort had dMMR, which was lower than rates quoted in other studies exploring dMMR status in sebaceous neoplasms (49–66%) (Table 1) [36,37,38]. The lower rate quoted by Cook et al. may be attributed to their focus on only sebaceous carcinomas instead of sebaceous neoplasms in general, as well as the inclusion of periocular tumors, which are typically MMR-proficient.

Studies have consistently shown that extraocular sebaceous neoplasias are more likely to be dMMR. Jessup et al. [38] and Cook et al. [2] performed the largest studies analyzing dMMR status and stratified groups by tumor location. Jessup et al. found that tumor location was significantly (*p* < 0.0001) associated with IHC status: 95% (*n* = 59/62) of non-head and neck tumors were dMMR compared to 55% (*n* = 84/154) of head and neck tumors. However, the authors noted that the sensitivity of tumor location to determine deficiency or proficiency of MMR was low. Cook et al. similarly found that most sebaceous carcinomas with dMMR were at sites other than the head and neck (65%), although the rates were much lower compared to Jessup et al. This study also investigated extraocular sebaceous carcinomas and found that dMMR tumors were most often located on the trunk (45%; *n* = 29/64) compared with MMR-proficient tumors (6%; *n* = 6/100) [2]. Although these studies have shown that dMMR is significantly associated with tumor location, solely relying on location to determine which tumors are potentially dMMR would miss many dMMR tumors, thus potentially decreasing their chances of identifying a germline variant in this population if one were to screen by location.

These studies also examined age of onset as a criterion for MMR-IHC testing. Jessup et al. [38] found a similar median age of tumor onset in individuals with dMMR status compared to those with MMR-proficient status in their sebaceous tumors, with the median ages at diagnosis being 68 and 66, respectively. Cook et al. [2] found that the median age of tumor onset was slightly younger in individuals whose tumors were dMMR versus MMR-proficient, with the median ages at diagnosis being 67 and 78, respectively; notably, this difference was not statistically significant. This variance in median age can likely be attributed to tumor location and pathologies evaluated in each study (Table 1) [2,38]. These studies indicate that utilizing age for tumor diagnosis, as well as tumor location, as an alternative for screening with MMR-IHC would likely miss a significant number of tumors suggestive of MTS.

Sebaceous neoplasms with dMMR most often demonstrated loss of *MSH2/MSH6* on MMR-IHC across all studies (60–74%), with loss of *MLH1/PMS2* (19–23%) as the second most common finding (Table 1). Jessup et al. [38] postulated that the highest frequency of *MSH2/MSH6* deficiency may reflect the fact that *MSH2* is the most common germline mutation in MTS, although germline mutation status was not confirmed in their cohort. Cook et al. [2] and Everett et al. [37] found that, of those who underwent MMR-IHC and germline testing, most pathogenic variants were detected in *MSH2* (Table 2). Schon et al. [36] evaluated germline mutation status in their cohort; however, due to the small sample size, only two pathogenic variants were identified, making it challenging to identify significant trends (Table 2). *MLH1* promoter methylation was not explored but would be reasonable to consider in future studies, given the known high prevalence of this finding in sporadic colorectal tumors with *MLH1/PMS2* deficiency. Germline testing was not performed for large portions of the cohorts for these studies; therefore, it is hard to conclusively state the use of MMR-IHC in deciding whether one should undergo germline testing. However, when dMMR is identified in a sebaceous neoplasm, this should prompt consideration for an associated pathogenic germline variant.

Although MMR-IHC has been explored in an unselected population of individuals with sebaceous neoplasias, a few studies have investigated MSI in sebaceous tumors outside of the context of MTS. Kruse et al. [39] examined 58 paraffin-embedded sebaceous gland tumors from 58 patients from Germany and Italy. Of these tumors, 25 were sebaceous gland neoplasms and 32 were sebaceous gland hyperplasias. An MSI assessment revealed that 60% (*n* = 15/25) of sebaceous gland neoplasms and 3% (*n* = 1/32) of sebaceous gland hyperplasias were MSI-H. A subsequent study showed that in a population suspicious of MTS due to having a personal history of a sebaceous neoplasm and at least one additional malignancy, or a personal history of a sebaceous neoplasm with a family history suspicious of Lynch syndrome, 65% (*n* = 24/37) of patients with MSI-H tumors were identified with a pathogenic variant consistent with a diagnosis of MTS [40]. Although MSI testing is typically considered in the context of a hereditary condition, such as Lynch syndrome, other etiologies for MSI in sebaceous neoplasms are important to consider. A small study investigated MSI in apparently sporadic sebaceous carcinomas and found a higher rate of MSI in immunosuppressed renal transplant recipients (60%, *n* = 3/5) compared to immunocompetent individuals (0%, *n* = 0/3). Given the relatively small sample size, the authors were unable to establish significant conclusions but postulated that chronic exposure to immunosuppressive drugs, such as azathioprine, could induce MSI due to the preferential selection of cells with dMMR as a mechanism to avoid cytotoxicity [41]. MSI is not routinely used as a stand-alone screening method for sebaceous neoplasms; however, if MSI is utilized for screening sebaceous neoplasms, it would be important to consider factors such as immunosuppression and dMMR when considering the etiology of MSI-H status.

MSI testing and MMR-IHC status have been well documented to have high concordance in colorectal tumors. In the limited studies available, MSI-H and dMMR of sebaceous neoplasms appear to have high concordance as well. IHC screening in sebaceous neoplasias is primarily performed in the context of evaluating for a hereditary predisposition but may find increasing use in consideration with systemic therapy for advanced disease. The yield of MSI and IHC screening in sebaceous carcinomas for germline mutations is challenging to determine due to the relatively small number of individuals who undergo MSI and dMMR tumor screening and germline genetic testing. As a result, although it may have use for certain patients, such as those with metastatic disease, universal screening for sebaceous neoplasms via MSI and dMMR for the purposes of determining good candidates for germline testing may be difficult to justify in practice.

## 5. Hereditary Cancer Predisposition Syndromes Associated with Sebaceous Neoplasms

Although most sebaceous neoplasms are sporadic in origin, it is known that they occur at higher frequencies in individuals with certain risk factors, including genetic predisposition. MTS has long been associated with sebaceous neoplasms and tumors of the gastrointestinal and genitourinary systems and is caused by mutations in DNA MMR genes, which lead to MSI. MTS was initially described separately by Muir and Torre in 1967, who characterized the classical features of the syndrome as the presence of a cutaneous sebaceous neoplasm(s) with one or more visceral neoplasms, of which colonic neoplasias are the most common in the absence of other causative factors [42,43]. MTS is an uncommon variant of Lynch syndrome, having an estimated prevalence of about 1 in 500,000 versus 1 in 279 individuals, respectively [2,44]. The proportion of patients with MTS has been estimated to be between about 5% and 9% of all Lynch syndrome patients and has a male-to-female predominance with a ratio of three males for every two females diagnosed [45]. Patients with this syndrome generally present between 53 and 61 years of age, with the earliest reported case at 23 years and the oldest at 89 years [46]. Although Lynch syndrome is associated with five genes affecting MMR—*MLH1*, *MSH2*, *MSH6*, *PMS2*, and *EPCAM*—to this point, MTS has been primarily associated with three of these genes: *MLH1*, *MSH2*, and *MSH6*. The majority of patients with MTS are due to pathogenic variants in the *MSH2* gene (88–93%), while *MLH1* is the next most common (7–11%) [40,47]. Pathogenic variants in *MSH6* and *PMS2* are the least common germline causes of MTS, with primarily single-case reports of patients and their families reported in the literature [36,48,49]. Elevated risks of sebaceous tumors and keratoacanthomas have not been reported for individuals with *EPCAM* deletions to date. At least 50% of patients diagnosed with MTS present with an internal malignant disease before skin lesions develop, of which colorectal cancer (CRC) is the most common. It is critical to identify these patients as early as possible to aid in proactive decision-making and facilitate education for their family members who may benefit from testing [50].

Currently, no consensus on the clinical diagnostic criteria for MTS has been published. However, personal medical history, including the presence of a cutaneous sebaceous neoplasm with one or more visceral neoplasms, in addition to other personal and familial factors, can be suggestive of MTS. As previously discussed, dMMR and MSI can be indicative of Lynch syndrome. Pathogenic variants in a Lynch syndrome-associated gene identified on somatic testing can also be suggestive of a germline mutation. In patients with dMMR colorectal tumors, there is an approximate 20–30% yield for germline testing for mutations associated with Lynch syndrome, which is consistent with the reported yield of germline testing for patients with sebaceous neoplasms with dMMR (20–50%) (Table 2) [2,36,37,51,52]. Given the similar yield for identifying a germline Lynch syndrome mutation in patients with dMMR in sebaceous carcinomas and CRC, it is reasonable to evaluate for germline testing in the presence of abnormal MMR-IHC testing. With regards to MSI, one study looked at 1066 patients with newly diagnosed colonic adenocarcinoma. Of these, 135 tumors (12.7%) were MSI-H, and 23 (17%) had a germline deleterious mutation [32]. MSI testing of sebaceous neoplasms in specific patients may have a high germline yield. Mangold et al. demonstrated that in a selected patient population, the yield for germline testing in individuals who had MSI-H tumors was about 65% [40]. Based on this result, it is important to assess individuals for signs and symptoms of MTS, for which a referral to a genetics specialist, such as a genetic counselor, may be invaluable.

In addition to somatic testing that may be suggestive of a germline mutation, family history can aid in determining the level of suspicion for MTS. Although sebaceous neoplasms can occur in isolation for individuals with MTS, they are often seen in combination with other cancers, affecting both the patient and their family members. As a result, a genetics consult in which family history is assessed and evaluated is invaluable. It has been estimated that sebaceous neoplasms precede the internal neoplasms or are concurrent with them in 41% of individuals with MTS [53]. Of the internal neoplasms, the risk is highest for colonic and endometrial cancers, with gastric, ovarian, pancreatic, urothelial, brain (typically glioblastoma), biliary tract, and small intestinal cancers also being associated with Lynch syndrome [49]. Features that raise suspicion of a germline variant within a family include the following:

- Lynch syndrome-associated cancer diagnosed <age 50;
- Synchronous or metachronous Lynch syndrome-associated cancers;
- Multiple relatives on the same side of the family with Lynch syndrome-associated cancers.

If a patient’s personal and/or family history is suspicious, identification of a germline pathogenic variant can impact the cancer treatment and the proactive management recommendations for individuals and their families. It is important to note that a family history suspicious for a hereditary cancer condition is an indication for referral to genetics services, regardless of the somatic testing results.

### Alternative Genetic Etiologies for MTS Phenotype

Although MSI can be indicative of an increased risk for MTS, it is estimated that approximately 35% of tumors in patients with this syndrome do not show instability [50]. In this case, the assumption is that the syndrome is not driven by errors within MMR proteins, as previously discussed, but rather an alternative mechanism. Multiple case reports have identified MTS patients with tumors that show MSS and biallelic inactivation of *MUTYH*, and their authors suggest MAP as an alternative etiology for MTS, separate from a variant of Lynch syndrome [3,4]. The function of *MUTYH* is in base excision repair, in which it removes adenine bases from the DNA backbone at sites where adenine is inappropriately paired. Biallelic inactivation of *MUTYH* in the germline due to an associated pathogenic variant causes MAP. MAP is a hereditary cancer predisposition syndrome associated with a significantly increased risk of colorectal polyps (typically between ten to a few hundred), with an average age of presentation of approximately 50 years old [54]. Unlike Lynch syndrome, which is inherited in an autosomal dominant manner, MAP is inherited in an autosomal recessive manner and does not display the familial patterns often seen in Lynch syndrome. When compared to MTS associated with dMMR, MTS tumors that are driven by biallelic *MUTYH* inactivation are often lower in penetrance and present at older ages than their MMR counterparts [55].

## 6. Recommendations and Management for Sebaceous Neoplasms

### 6.1. Somatic Testing of Sebaceous Neoplasms and Germline Testing After Sebaceous Neoplasm Diagnosis

As dMMR has been shown to be a good Lynch syndrome predictor across a broad spectrum of tumor types [56], it is tempting to perform MMR-IHC on all sebaceous neoplasms to identify individuals at high risk for Lynch syndrome. However, due to a high false-positive rate of MMR-IHC (56%) in sebaceous neoplasms, this is unlikely to be effective as a single method for identifying those with Lynch syndrome. Of note, MMR-IHC may be beneficial for reasons other than identifying Lynch syndrome, including assessing for potential response to immunotherapy in advanced diseases [57].

The sensitivity and specificity of MMR-IHC on sebaceous neoplasms for Lynch syndrome are lower than those of CRC and endometrial cancer [58], and, therefore, MMR-IHC is less likely to accurately determine if an individual is at high risk to have Lynch syndrome [57]. The correlation between sebaceous neoplasms with dMMR and a germline Lynch syndrome mutation was highest when patients had either a personal history of CRC and/or a family history of two or more relatives with CRC [58]. Therefore, additional Lynch syndrome features are a better predictor of Lynch syndrome diagnosis than MMR deficiency of a sebaceous neoplasm, and MMR-IHC on all sebaceous neoplasms may not be necessary.

Although several tools are available to identify individuals with CRC at high risk for Lynch syndrome, only one such tool exists to address this question in individuals with sebaceous neoplasms. The Mayo Muir–Torre syndrome risk scoring system provides a score based on the age at diagnosis of a sebaceous neoplasm, the total number of sebaceous neoplasms, personal history of a Lynch syndrome-associated cancer, and family history of any Lynch syndrome-associated cancer. The creators of the scoring system found that individuals with a score of 3 or higher were more likely to have MTS, individuals with a score of 2 had an intermediate likelihood, and no individual with a score of 1 or less had MTS [57].

**Figure 1:**
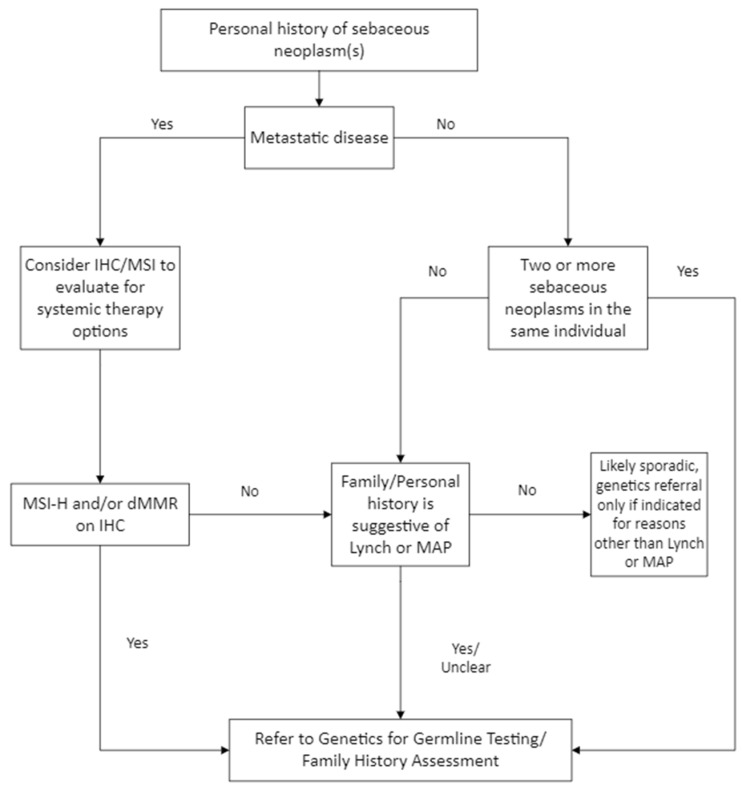
When a genetics referral is indicated for a patient with sebaceous neoplasm(s).

Overall, any individual with a sebaceous neoplasm should be screened for Lynch syndrome, including an assessment for additional Lynch syndrome features, such as personal and family history of Lynch syndrome-related cancers (Figure 1). If additional Lynch syndrome features are present that meet a score of 2 or higher on the Mayo scoring tool, referral to genetics for germline testing is indicated (Table A1). Prior to germline testing, if an individual had CRC or endometrial cancer, performing MMR-IHC on these tumors may provide additional insight into the possibility of Lynch syndrome. This is because the specificity of MMR-IHC on CRC and endometrial cancer is much higher than that on sebaceous neoplasms.

MMR-IHC on sebaceous neoplasms should not be used in isolation for the identification of candidates for germline testing. The Mayo scoring system indicates the importance of using the Lynch syndrome features of the personal and family history in addition to the personal history of sebaceous neoplasms to guide referrals to genetics for germline testing rather than MMR-IHC on sebaceous neoplasms. Germline genetic testing needs to include the Lynch syndrome-associated genes (*MLH1*, *MSH2*, *MSH6*, *PMS2*, *EPCAM*), as well as *MUTYH*. Germline multi-gene panels, including these genes, are currently commercially available. Identifying patients with Lynch syndrome or MAP can lead to increased cancer screening and potential chemopreventive options for the individual, as well as their relatives.

### 6.2. Lynch Syndrome and MAP Management

Multiple resources review guidelines for the screening and treatment of the many cancers associated with Lynch syndrome. The National Comprehensive Cancer Network (NCCN) guidelines are most often referenced for cancer screening recommendations in individuals with Lynch syndrome and MAP. The latest NCCN guidelines can be accessed here [https://www.nccn.org/guidelines/guidelines-detail?category=2&id=1544, accessed on 27 December 2024]. Cancer risk estimates and associated screenings vary by Lynch syndrome-associated genes and are updated regularly.

High-quality colonoscopy, typically recommended to be performed every 1–2 years, is the preferred colon screening method for all individuals with Lynch syndrome and MAP. The age at which to start screening varies by the genes affected, as well as family history, but may be initiated as early as age 20. Screening for gynecologic cancers, such as ovarian and endometrial cancers, remains controversial; transvaginal ultrasound and CA-125 have been found to have poor sensitivity and specificity but can be performed for women who have not undergone prophylactic hysterectomy/salpingo-oophorectomy. In addition, regular endometrial biopsy in asymptomatic women can be considered but has not been shown to be more effective than monitoring for abnormal bleeding suggestive of endometrial cancer. Gastric and small bowel cancer screening is performed using high-quality esophagogastroduodenoscopy (EGD), usually performed every 3–5 years in adults.

Limited data support pancreatic cancer screening in Lynch syndrome. Pancreatic cancer screening can be considered for individuals with at least one first- or second-degree relative with exocrine pancreatic cancer on the same side of the family as Lynch syndrome. Screening can start at age 50 or 10 years younger than the earliest exocrine pancreatic cancer diagnosis in the family, whichever comes first. The NCCN recommends pancreatic cancer screening to be performed in experienced high-volume centers, as there are uncertainties about the potential benefits of pancreatic cancer screening. Screening for other Lynch syndrome-associated cancers, such as those of the urinary tract, brain, or biliary tract, can be considered based on family history and personal risk factors as well.

Colon cancer screening for MAP includes high-quality colonoscopy every 1–2 years starting at age 25–30. If the polyp burden cannot be managed endoscopically, colectomy is recommended to reduce the colon cancer risk. Baseline EGD is recommended to begin at age 30–35. Cancer screening recommendations for both MAP and Lynch syndrome are updated regularly, so involvement of a genetics professional in managing these disorders is highly recommended [49].

## 7. Treatment of Sebaceous Carcinomas

According to a large survey from the National Cancer Database, over 95% (*n* = 1320/1388) of patients with sebaceous carcinomas are diagnosed with localized disease without nodal or distant metastases [59]. Surgical resection, whether by wide local excision or Mohs, remains the mainstay of treatment for most sebaceous carcinomas. Sentinel lymph node biopsy may be performed but is controversial due to concerns about low yield and false-negative results in the head and neck [59,60]. In addition, radiation and topical therapies have been used anecdotally in those with locally advanced disease [61,62,63,64]. For patients with locally advanced or metastatic disease, systemic therapy has been used in the neoadjuvant or palliative setting. 5-Fluorouracil/cisplatin, 5-fluorouracil/carboplatin, mitomycin-C, and cetuximab in combination with radiation have been explored in this area [65,66,67,68]. However, with the advent of immunotherapy and the discovery that dMMR tumors are more responsive to immunotherapy, checkpoint inhibitors have started to be used with good results. In 2018, Domingo-Musibay et al. reported a nearly complete response to pembrolizumab for a patient with a widely metastatic sebaceous carcinoma [69]. Although this tumor was MSS, it had high expression of PDL-1. Subsequent case reports have confirmed the efficacy of pembrolizumab, either alone or in combination with carboplatin, in the metastatic setting [70,71,72]. Of interest, one of these carcinomas was responsive to immunotherapy, despite being MSS and having a PDL-1 expression of <1%. Based on these results, immunotherapy for those with advanced sebaceous carcinomas may be effective for those with tumors that are MSI, have a high expression of PDL-1, or have a high mutational burden [72]. These tests are recommended for all patients with metastatic disease. In addition, neoadjuvant use of immunotherapy is now being explored in order to prevent exenteration and allow for less extensive surgery for those with locally advanced disease [73].

## 8. Conclusions/Future Directions

Current knowledge regarding somatic and germline changes in sebaceous neoplasms has provided valuable information regarding the etiology of many of these tumors. In turn, these insights are starting to guide decisions regarding systemic therapy in patients with advanced disease, as well as aiding in the identification of patients and families at risk of internal malignancies associated with hereditary cancer conditions, such as Lynch syndrome. However, the use of somatic testing, such as MMR-IHC or MSI, as sole assessments for hereditary risk is suboptimal, and reviews of the personal and family histories of cancer and colon polyps are critical for all patients with sebaceous neoplasms. In addition, questions remain about the interactions between somatic profiles, such as those associated with UV radiation and immunosuppression, and germline mutations in this setting. As these pathways become better understood, they have the potential to provide further guidance for the treatment and management of individuals with Lynch syndrome and MUYTH-associated polyposis, as well as those with sporadic sebaceous neoplasms.

## Figures and Tables

**Table 1:** Yield of dMMR and MSI testing in sebaceous neoplasms.

	Everett et al., 2014 (*n* = 77) [37]	Jessup et al., 2016 (*n* = 216) [38]	Schon et al., 2018 (*n* = 53) [36]	Cook et al., 2023 (*n* = 220) [2]
dMMR	38/77 (49%)	143/216 (66%)	26/53 (49%)	70/220 (32%)
Extraocular	Not specified	216 (100%)	Not specified ^a^	64/164 (39%)
Periocular	Not specified	N/A	Not specified ^a^	6/56 (11%)
Head and Neck SN with dMMR	Not specified	84/154 (55%)	Not specified	24/104 (30%) ^a,b^
Non-Head and Neck SN with dMMR	Not specified	59/62 (95%)	Not specified	36/55 (65%) ^a,b^
dMMR results on IHC				
*MSH2/MSH6*	27/38 (71%) (*MSH2* or *MSH2/MSH6*)	90/143 (60%)	19/26 (73%)	52/70 (74.3%)
*MLH1/PMS2*	9/38 (23%) (*MLH1* or *MLH1/PMS2*)	27/143 (19%)	6/26 (23%)	15/70 (21.4%)
*MSH6*	2/38 (5%) ^c^	22/143 (15%)	1/26 (3.8%)	2/70 (2.9%)
*PMS2*	0 (0%)	4/143 (3%)	0 (0%)	1/70 (1.4%)
MSI				
MSI-H	10/40 ^d^	Not evaluated	Not evaluated	Not evaluated

SN = sebaceous neoplasm. ^a.^ Not specified if extraocular or periocular; 51/71 were head and neck. ^b.^ Inclusion criteria for this study involved patients with sebaceous carcinomas. ^c.^ Equivocal or weak staining shown for these patients. ^d.^ MSI testing could not be completed in 37 (40.5%) SN tumors due to insufficient samples. All MSI-H tumors had dMMR (mostly *MSH2/MSH6*).

**Table 2:** Germline mutation status in individuals with sebaceous neoplasms.

	Everett et al., 2014 ^a^ [37]	Jessup et al., 2016 [38]	Schon et al., 2018 [36]	Cook et al., 2023 ^b^ [2]
Patients who underwent germline testing	86	Not analyzed	10	56
*MLH1* PV	5 (5.8%)		0 (0%)	3 (5.4%)
*MSH2* PV	18 (20.9%)		1 (1%)	24 (42.9%)
*MSH6* PV	1 (1.2%		0 (0%)	3 (5.4%)
*PMS2* PV	1 (1.2%)		1 (1%)	0 (0%)
VUS	2 (2.3%) ^c^		1 (1%) ^d^	0 (0%)
Negative	59 (68.6%)		7 (70%)	26 (46.4%)

PV = pathogenic variant; VUS = variant of uncertain significance. ^a.^ 77/86 had IHC testing on their SN. Reason testing of SN was not completed includes: known MMR mutations in the family (3/9), tumor testing performed on a different Lynch syndrome-associated tumor (4/9), and family history meeting Amsterdam I/II clinical diagnostic criteria, warranting direct germline genetic testing (2/9). ^b.^ Inclusion criteria for this study involved patients with sebaceous carcinomas. ^c.^ Results in MSH2 and MSH6. ^d.^ VUS in *MSH6*, loss of *MLH1*, and *PMS2* on IHC.

## Data Availability

No new data were created or analyzed in this study. Data sharing is not applicable to this article.

## Abbreviations

The following abbreviations are used in this manuscript:

CRC	Colorectal Cancer
EGD	Esophagogastroduodenoscopy
HPV	Human Papillomavirus
IHC	Immunohistochemistry
MAP	MUTYH-Associated Polyposis
MMR	Mismatch Repair
MSI	Microsatellite Instability
MSI-H	Microsatellite Instability High
MSI-L	Microsatellite Instability Low
MSS	Microsatellite Stable
MTS	Muir–Torre Syndrome
NCCN	National Comprehensive Cancer Network
UV	Ultraviolet

## Appendix A

**Table A1:** Genetic Testing Guidelines for Sebaceous Carcinoma.

NCCN Lynch Syndrome (2024) [49]	Mayo MTS Risk Scoring System (2014) [57]
**Personal History**	
- Known LS PV in the family - Personal history of an LS-related cancer ^a^ and any of the following: Diagnosed <50 A synchronous or metachronous LS-related cancer ^a^ regardless of age 1 First-degree or second-degree relative with an LS-related cancer ^a^ diagnosed <50 ≥2 First-degree or second-degree relatives with an LS-related cancer ^a^ regardless of age	- Two or more sebaceous neoplasms OR - At least two of the following: Sebaceous neoplasm younger than 60 Personal history of an additional LS-related cancer ^b^ Family history * of any LS-related cancer ^b^ * Does not specify degree of relations Score of 2 or more, 100% sensitivity, and 81% specificity for predicting a germline mutation in an LS MMR gene
Family History (Unaffected Proband)	
≥1 First-degree relative with a CRC or EC diagnosed <50 ≥1 First-degree relative with a CRC or EC and a synchronous or metachronous LS-related cancer ^a^ regardless of age ≥2 First-degree or second-degree relatives with LS-related cancers ^a^ including ≥1 diagnosed <50 ≥3 First-degree or second-degree relatives with LS-related cancers ^a^ regardless of age	Must be affected to meet criteria for testing.

LS = Lynch syndrome. ^a^. Colorectal, endometrial, gastric, ovarian, pancreatic, urothelial, brain (usually glioblastoma), biliary tract, small intestine, sebaceous adenomas, sebaceous carcinomas, and keratoacanthomas. ^b.^ Colorectal, endometrial, ovarian, small bowel, urinary tract, and biliary tract cancers.
